# Dynamic Organelle Remodeling in HIV-Associated Myocardial Disease: Mechanisms, Fibrotic Pathways, and Therapeutic Opportunities

**DOI:** 10.3390/cimb48040371

**Published:** 2026-04-02

**Authors:** Katongo Hope Mutengo, Sepiso Kenias Masenga, Annet Kirabo

**Affiliations:** 1 Department of Internal Medicine, Ministry of Health, Monze Mission Hospital, Monze 10101, Zambia; hope.mutengo@moh.gov.zm; 2 Department of Internal Medicine, School of Medicine, University of Zambia, Lusaka 10101, Zambia; 3 Department of Medicine, Division of Clinical Pharmacology, Vanderbilt University Medical Center, Nashville, TN 37235, USA; sepisomasenga@lcpts.org; 4 Department of Pathology, Mulungushi University, Livingstone 10101, Zambia; 5 Department of Molecular Physiology and Biophysics, Vanderbilt University Medical Center, Nashville, TN 37235, USA

**Keywords:** organelle, mitochondrial, HIV, myocardial, fibrosis, remodeling

## Abstract

People with HIV experience a disproportionate burden of myocardial fibrosis and diastolic dysfunction that is not fully explained by traditional cardiovascular risk factors or systemic inflammation. Emerging evidence suggests that HIV-associated cardiomyopathy originates from persistent disturbances in cardiomyocyte homeostasis driven by chronic immune-metabolic stress. Metabolic dysregulation, antiretroviral-related toxicity, and residual inflammatory signaling converge at the cardiomyocyte organelle level, leading to mitochondrial dysfunction, endoplasmic reticulum stress, and impaired autophagy. These interrelated processes precede overt structural heart disease and promote progressive myocardial stiffening, despite effective viral suppression. Framing myocardial remodeling as a consequence of unresolved organelle stress highlights opportunities for earlier intervention, including aggressive management of metabolic risk factors, the use of established cardioprotective therapies with antifibrotic effects, and emerging strategies targeting mitochondrial and proteostatic pathways. This organelle-centered perspective supports prevention-focused approaches that combine accessible imaging modalities and circulating biomarkers to mitigate the long-term cardiovascular risk in people with HIV, particularly in resource-limited settings.

## 1. Introduction

The cardiovascular complications observed in people with HIV cannot be fully explained by traditional risk factors or systemic inflammation alone [1,2]. Increasing evidence suggests that myocardial injury in treated HIV is rooted in persistent disturbances of cellular homeostasis, particularly within cardiomyocytes that are chronically exposed to immune-metabolic stress [3,4]. Long-standing dyslipidemia, insulin resistance, and residual inflammatory signaling impose a continuous energetic and proteostatic burden on cardiomyocyte organelles, subtly reshaping intracellular function long before overt cardiac disease becomes clinically apparent [4,5,6,7].

Rather than presenting as acute myocardial injury, HIV-associated cardiac remodeling appears to evolve through gradual subcellular maladaptation [6]. Mitochondria experience sustained metabolic overload and oxidative stress; the endoplasmic reticulum (ER) struggles to maintain protein folding and calcium balance; and lysosomal–autophagy pathways become insufficient to clear damaged components [3,6,7]. As these stress responses persist, their effects become increasingly interconnected. Mitochondrial dysfunction amplifies reactive oxygen species (ROS) generation and energetic insufficiency; ER stress perturbs calcium handling and proteostasis; and defective lysosomal clearance allows damaged organelles and proteins to accumulate [7,8,9]. Together, these maladaptive processes converge on canonical fibrotic pathways—such as Transforming Growth Factor Beta 1 (TGF-β1), Nuclear Factor kappa-light-chain-enhancer of activated B cells (NF-κB), and Hippo signaling with Yes-Associated Protein and Transcriptional co-Activator with PDZ-binding motif (YAP/TAZ)—culminating in extracellular matrix expansion and progressive myocardial stiffening [10,11,12].

Despite accumulating evidence of systemic metabolic and inflammatory injury in HIV, the organelle-level mechanisms linking these upstream perturbations to myocardial fibrosis remain poorly integrated in the current literature. Existing reviews have largely examined metabolic complications or oxidative stress as isolated processes, with comparatively limited emphasis on how coordinated interactions among mitochondria, the ER, and lysosomes drive the transition from cellular stress to tissue-level fibrosis. Moreover, the translational potential of targeting organelle homeostasis through metabolic modulation, antioxidant strategies, or enhancement of autophagic flux has not been fully synthesized within the context of HIV-associated metabolic disease.

This review therefore aims to synthesize mechanistic and translational evidence on how HIV-related metabolic disturbances and chronic immune activation remodel subcellular organelles within cardiomyocytes. We examine how mitochondrial, ER, and lysosomal dysfunction intersect to promote profibrotic signaling, and we discuss emerging therapeutic strategies that may restore organelle homeostasis and mitigate myocardial fibrosis. By framing cardiac remodeling as a consequence of dynamic organelle cross-talk, this review provides a conceptual and practical basis for developing organelle-targeted interventions for HIV-associated cardiometabolic disease in both high- and low-resource settings. The key knowledge gaps and corresponding conceptual contributions of this organelle-centered framework are summarized in Table 1.

## 2. Subcellular Origins of Myocardial Fibrosis in HIV

### 2.1. Why Fibrosis Begins Inside the Cardiomyocyte

Myocardial fibrosis does not arise abruptly at the tissue level; rather, it develops gradually as cardiomyocytes adapt—and eventually maladapt—to chronic metabolic and inflammatory stress [4]. In people with HIV, long-standing dyslipidemia, insulin resistance, and immune activation continuously challenge cellular homeostasis [4,5,6]. Cardiomyocytes, which rely heavily on coordinated organelle function to sustain contractility and survival, respond to this stress first at the subcellular level [13,14]. At this early stage, fibrosis is not yet visible on imaging or reflected in overt functional impairment. Instead, a sequence of intracellular events unfolds across mitochondria, ER, and the lysosomal–autophagy pathways, progressively shifting cardiomyocytes toward a profibrotic state.

The following sections examine how mitochondrial, ER, and lysosomal dysfunction individually and collectively drive myocardial fibrosis in HIV.

### 2.2. Mitochondrial Dysfunction in HIV: Metabolic Overload as a Trigger for Oxidative and Profibrotic Signaling

**Figure 1:**
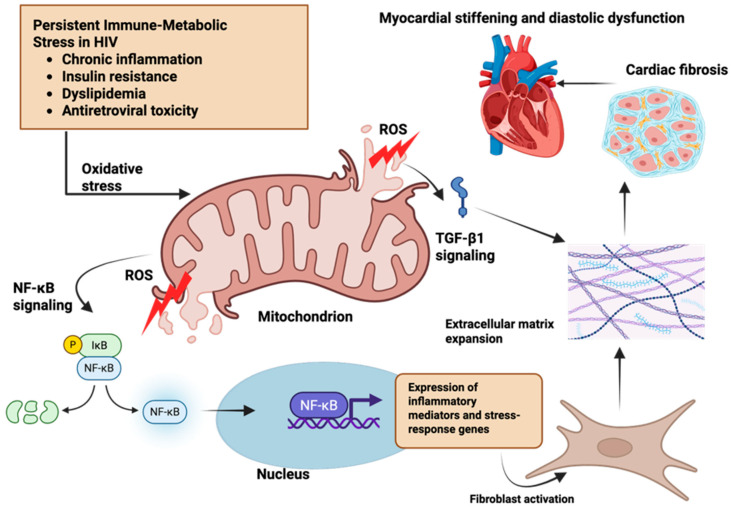
Immune-metabolic stress-driven mitochondrial signaling links HIV to myocardial fibrosis. Persistent immune-metabolic stress in people with HIV including chronic inflammation, insulin resistance, dyslipidemia, and antiretroviral toxicity, induces mitochondrial dysfunction in cardiomyocytes. Mitochondrial injury and reactive oxygen species (ROS) generation activate redox-sensitive signaling pathways, particularly nuclear factor kappa B (NF-κB), leading to nuclear translocation and transcription of inflammatory and stress-response genes. These signals promote fibroblast activation and extracellular matrix remodeling, culminating in myocardial fibrosis and progressive tissue stiffening. Created in BioRender. Masenga, S. (2026) https://BioRender.com/70598yb.

The accumulation of oxidative mitochondrial injury has important signaling consequences because mitochondrial ROS do more than cause cellular damage—they actively function as signaling molecules [7,8]. In cardiomyocytes exposed to chronic immune-metabolic stress in HIV, sustained ROS production alters intracellular signaling pathways that regulate inflammation, stress responses, and tissue remodeling [4,15] (Figure 1). One key pathway activated by mitochondrial ROS is NF-κB signaling. Under conditions of oxidative stress, ROS promote the activation of upstream kinases that release NF-κB from its inhibitory complex, allowing it to enter the nucleus [8]. Once activated, NF-κB drives the expression of inflammatory mediators and stress-response genes that sustain a low-grade inflammatory state within the myocardium. In people with HIV, where baseline immune activation often persists despite viral suppression, this ROS-dependent activation of NF-κB further amplifies inflammatory signaling within the cardiomyocytes and surrounding cardiac tissue.

In parallel, mitochondrial oxidative stress enhances TGF-β1 signaling, a central driver of fibrosis. ROS facilitate the activation of latent TGF-β1 stored in the extracellular matrix and strengthen downstream transcriptional responses that promote collagen synthesis and extracellular matrix accumulation [10,16]. In the HIV setting, this profibrotic bias is reinforced by chronic inflammatory cues and metabolic stress, which sensitize cardiac cells to TGF-β1-mediated remodeling even in the absence of overt myocardial injury [6,17]. Importantly, mitochondrial dysfunction in HIV does not remain confined within the cardiomyocyte. When mitophagy is impaired, damaged mitochondria release mitochondrial DNA (mtDNA) and other mitochondrial-derived danger signals into the cytosol and extracellular space [7,13,18,19]. These signals engage innate immune sensing pathways, further reinforcing inflammatory signaling and creating a feed-forward loop in which inflammation worsens mitochondrial injury, and mitochondrial injury sustains inflammation. This loop is particularly relevant in HIV, where immune activation is chronic and resolution mechanisms are often incomplete.

Through these combined processes, mitochondrial dysfunction becomes an early intracellular hub linking immune-metabolic stress to fibrotic remodeling. Cardiomyocytes under persistent oxidative and energetic stress generate paracrine signals that promote fibroblast activation and extracellular matrix deposition, leading to progressive myocardial stiffening [20,21,22]. Over time, these subclinical changes accumulate, providing a mechanistic explanation for the high burden of myocardial fibrosis and diastolic dysfunction observed in people with HIV, even in the absence of advanced atherosclerosis or uncontrolled viremia [23,24].

The coordinated mitochondrial mechanisms that initiate this transition from metabolic stress to profibrotic remodeling in HIV are summarized in Figure 1. 

Importantly, mitochondrial dysfunction does not remain isolated but propagates stress to other organelles, particularly the ER and lysosomal–autophagy system, establishing the basis for an interconnected organelle stress network.

### 2.3. Endoplasmic Reticulum Stress: How Disrupted Proteostasis Amplifies Profibrotic Signaling in HIV

**Figure 2:**
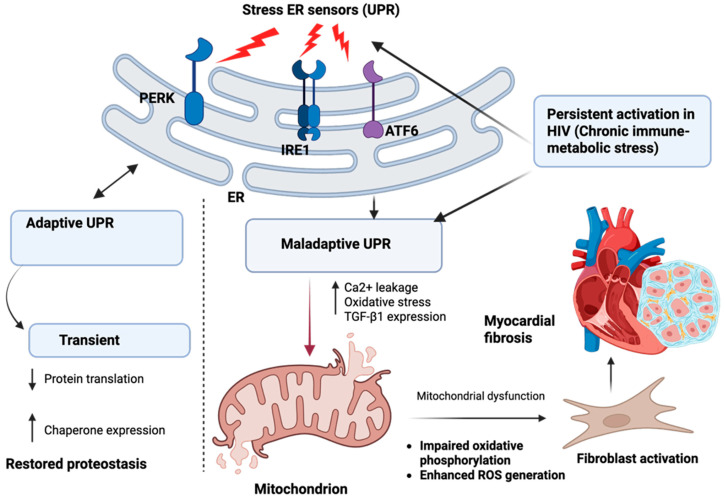
Persistent endoplasmic reticulum (ER) stress drives maladaptive unfolded protein response (UPR) signaling and promotes myocardial fibrosis in HIV. Chronic immune-metabolic stress in HIV leads to sustained activation of ER stress sensors: protein kinase RNA-like endoplasmic reticulum kinase (PERK), inositol-requiring enzyme 1 (IRE1), and activating transcription factor 6 (ATF6). While transient activation of these pathways supports adaptive proteostasis, persistent stimulation shifts the UPR toward a maladaptive state. Maladaptive UPR signaling disrupts calcium homeostasis and exacerbates mitochondrial dysfunction, amplifying oxidative stress and promoting downstream profibrotic signaling. These processes facilitate fibroblast activation, extracellular matrix accumulation, and progressive myocardial fibrosis. These effects extend beyond the ER itself, as disrupted calcium handling and proteostasis further amplify mitochondrial dysfunction and impair downstream autophagic clearance, reinforcing organelle cross-talk. Created in BioRender. Masenga, S. (2026) https://BioRender.com/550vswe.

The ER plays a central role in maintaining cardiomyocyte homeostasis by coordinating protein folding, lipid synthesis, and intracellular calcium storage. As highlighted earlier, in people with HIV, chronic immune-metabolic stress—driven by dyslipidemia, insulin resistance, and persistent inflammatory signaling—places a sustained burden on ER function [25]. Increased lipid flux, oxidative stress, and inflammatory mediators disrupt normal protein folding within the ER lumen, leading to the accumulation of misfolded or unfolded proteins and the activation of the unfolded protein response (UPR) [13,26,27]. In its early phase, UPR activation is adaptive. By transiently attenuating global protein translation and upregulating molecular chaperones, the ER attempts to restore proteostasis and limit further cellular injury [26,27]. However, in the setting of ongoing metabolic overload and unresolved inflammation characteristic of treated HIV, UPR signaling becomes prolonged and maladaptive (Figure 2). Persistent activation of the UPR sensors—protein kinase RNA-like endoplasmic reticulum kinase (PERK), inositol-requiring enzyme 1 (IRE1), and activating transcription factor 6 (ATF6)—shifts the UPR from an adaptive response toward one that promotes cellular dysfunction [27]. PERK, IRE1, and ATF6 are stress-sensing proteins embedded in the membrane of the ER that detect the accumulation of misfolded or unfolded proteins within the ER lumen [27,28]. When the ER protein-folding capacity is exceeded, these sensors become activated and initiate the UPR by reducing new protein synthesis, enhancing chaperone production, and coordinating adaptive signaling to restore proteostasis [13,26,27,28].

A key consequence of chronic ER stress is the disruption of calcium homeostasis. Sustained UPR activation increases calcium leakage from the ER into the cytosol, elevating intracellular calcium levels and placing a secondary burden on mitochondria (Figure 2). Excess mitochondrial calcium impairs oxidative phosphorylation, enhances ROS generation, and further exacerbates mitochondrial dysfunction [9,13,26]. In this way, ER stress and mitochondrial injury become tightly coupled, reinforcing intracellular stress through bidirectional organelle cross-talk.

Beyond its effects on cellular energetics, prolonged ER stress directly promotes profibrotic signaling [8,9,29]. Chronic activation of UPR pathways enhances TGF-β1 expression and sensitizes cardiomyocytes and resident fibroblasts to profibrotic cues [16,30]. This shifts the myocardial environment toward increased collagen synthesis, extracellular matrix accumulation, and reduced matrix turnover. Thus, ER stress in HIV is not merely a downstream marker of cellular injury but an active driver of fibrotic remodeling.

Together, these processes position ER dysfunction as a critical intermediary between immune-metabolic stress and myocardial fibrosis in HIV. By linking impaired proteostasis and calcium handling to mitochondrial dysfunction and profibrotic transcriptional programs, ER stress contributes to the gradual transition from adaptive cellular responses to maladaptive remodeling long before overt cardiac dysfunction becomes clinically apparent.

### 2.4. Lysosomal–Autophagy Dysfunction: Failure of Cellular Quality Control in HIV

**Figure 3:**
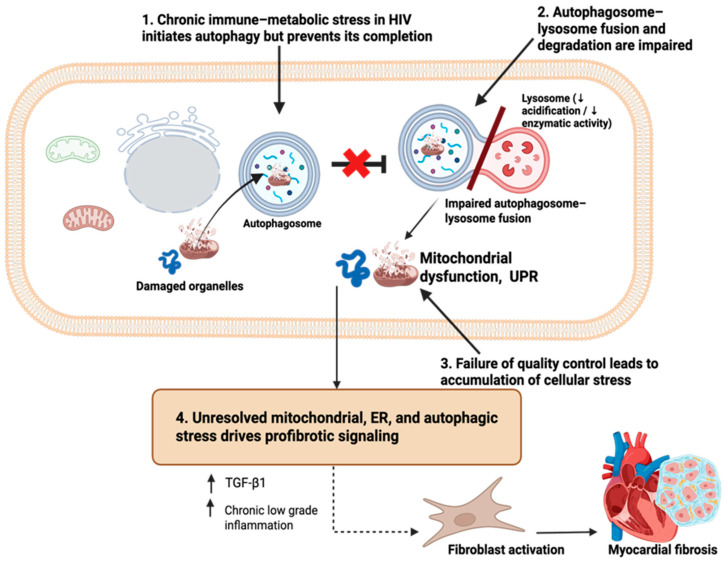
Impaired autophagic flux links unresolved organelle stress to myocardial fibrosis in HIV. Chronic immune-metabolic stress in people with HIV disrupts intracellular quality-control mechanisms within cardiomyocytes. Under physiological conditions, damaged mitochondria and misfolded proteins are sequestered into autophagosomes and delivered to lysosomes for degradation. In HIV-associated cardiometabolic disease, autophagosome fusion with lysosomes and subsequent enzymatic degradation are impaired. This defect in autophagic flux leads to the accumulation of dysfunctional mitochondria, unstable proteins, and oxidative by-products, sustaining mitochondrial and endoplasmic reticulum (ER) stress, with persistent activation of the unfolded protein response (UPR). Persistent organelle dysfunction activates profibrotic signaling pathways, promoting fibroblast activation, extracellular matrix deposition, and progressive myocardial fibrosis. As a result, failure of autophagic resolution prevents recovery from mitochondrial and ER stress, locking cardiomyocytes into a state of persistent intracellular injury that culminates in coordinated profibrotic remodeling. Created in BioRender. Masenga, S. (2026) https://BioRender.com/j7yn8e5.

Autophagy is the primary intracellular quality-control system responsible for the removal of damaged organelles, misfolded proteins, and other cellular debris [31]. In cardiomyocytes, this process is particularly important because of the high metabolic demand and limited regenerative capacity of these cells [32]. Autophagy proceeds through the sequestration of damaged cellular components into autophagosomes, which subsequently fuse with lysosomes where enzymatic degradation occurs in an acidic environment [31] (Figure 3). Together, efficient autophagosome formation, lysosomal function, and autophagic flux are essential for maintaining cardiomyocyte homeostasis under conditions of stress.

In people with HIV, multiple factors converge to impair this system. Chronic immune-metabolic stress, nutrient excess, insulin resistance, and antiretroviral therapy-related toxicity might disrupt both autophagosome formation and lysosomal function [3,33,34]. Inflammatory signaling and oxidative stress further interfere with lysosomal acidification and enzyme activity, reducing the capacity of lysosomes to degrade autophagic cargo [34,35]. As a result, autophagic flux becomes inefficient, even when autophagy is initially activated in response to cellular stress (Figure 3).

When autophagic clearance is impaired, damaged mitochondria and misfolded proteins accumulate within cardiomyocytes. These dysfunctional components continue to generate ROS and release mitochondrial-derived danger signals, sustaining inflammatory and stress signaling within the cell [32]. Importantly, the failure to remove damaged mitochondria prevents the resolution of mitochondrial oxidative stress and perpetuates the energetic and redox imbalance established earlier in HIV-associated metabolic disease [6,8].

Lysosomal–autophagy dysfunction also amplifies ER stress. Accumulation of misfolded proteins increases the burden on the ER, reinforcing UPR activation and calcium dysregulation [8,26,28,29]. In this way, impaired autophagy acts as a critical bottleneck that links mitochondrial dysfunction and ER stress, preventing recovery from adaptive stress responses and promoting a transition toward chronic cellular injury.

Beyond its role in stress amplification, defective autophagy directly contributes to profibrotic remodeling. Persistent intracellular stress enhances TGF-β1 signaling and sensitizes cardiomyocytes and resident fibroblasts to fibrotic cues [36]. In parallel, impaired clearance of damaged cellular components sustains low-grade inflammatory signaling, further reinforcing fibroblast activation and extracellular matrix deposition [16,36] Over time, this combination of unresolved organelle stress and persistent profibrotic signaling drives progressive myocardial stiffening.

Thus, lysosomal–autophagy dysfunction represents a key mechanism through which immune-metabolic stress in HIV is converted from a potentially reversible cellular insult into sustained fibrotic remodeling. By failing to resolve mitochondrial and ER stress, impaired autophagic clearance allows subcellular injury to accumulate and propagate, positioning autophagy failure as a central driver of myocardial fibrosis in people with HIV.

### 2.5. Convergence of Organelle Stress on Profibrotic Remodeling in HIV

The processes described above do not occur in isolation. Instead, mitochondrial dysfunction, ER stress, and impaired autophagic clearance form a tightly integrated organelle stress network in which injury in one compartment amplifies dysfunction in the others. This coordinated cross-talk transforms initially adaptive cellular responses into a sustained profibrotic state, providing a unifying framework for myocardial remodeling in HIV.

Chronic immune-metabolic stress in HIV does not injure mitochondria, the ER, or the autophagy–lysosomal system in isolation. Rather, these organelles form an interdependent stress network in which dysfunction in one compartment amplifies injury in the others [3,6,8,16,33]. Over time, this unresolved organelle cross-talk shifts cardiomyocytes from adaptive stress responses toward sustained profibrotic signaling [10,12]. Mitochondrial dysfunction often represents an initiating event. Excess lipid delivery, insulin resistance, and antiretroviral-related toxicity increase mitochondrial ROS production and impair oxidative phosphorylation [5]. Beyond energetic insufficiency, mitochondrial oxidative stress alters intracellular calcium handling and redox balance, placing a secondary burden on the ER [9,28]. Calcium leakage from the ER further exacerbates mitochondrial dysfunction, reinforcing a self-sustaining cycle of oxidative and metabolic stress.

In parallel, prolonged ER stress amplifies this process. Persistent activation of UPR signaling disrupts proteostasis and calcium homeostasis, sensitizing cardiomyocytes to inflammatory and profibrotic cues [7,13,26]. Importantly, ER stress enhances responsiveness to profibrotic mediators and promotes transcriptional programs that favor extracellular matrix synthesis rather than cellular recovery.

Under physiological conditions, autophagy serves as the resolution arm of this stress response by removing damaged mitochondria and misfolded proteins [31]. In HIV-associated metabolic disease, however, autophagic clearance becomes inefficient. While autophagosome formation may remain active, impaired fusion with lysosomes and reduced lysosomal degradative capacity prevent effective clearance [3,4,35]. As a result, damaged mitochondria and protein aggregates persist within cardiomyocytes, continuing to generate oxidative stress and danger-associated signaling.

**Figure 4:**
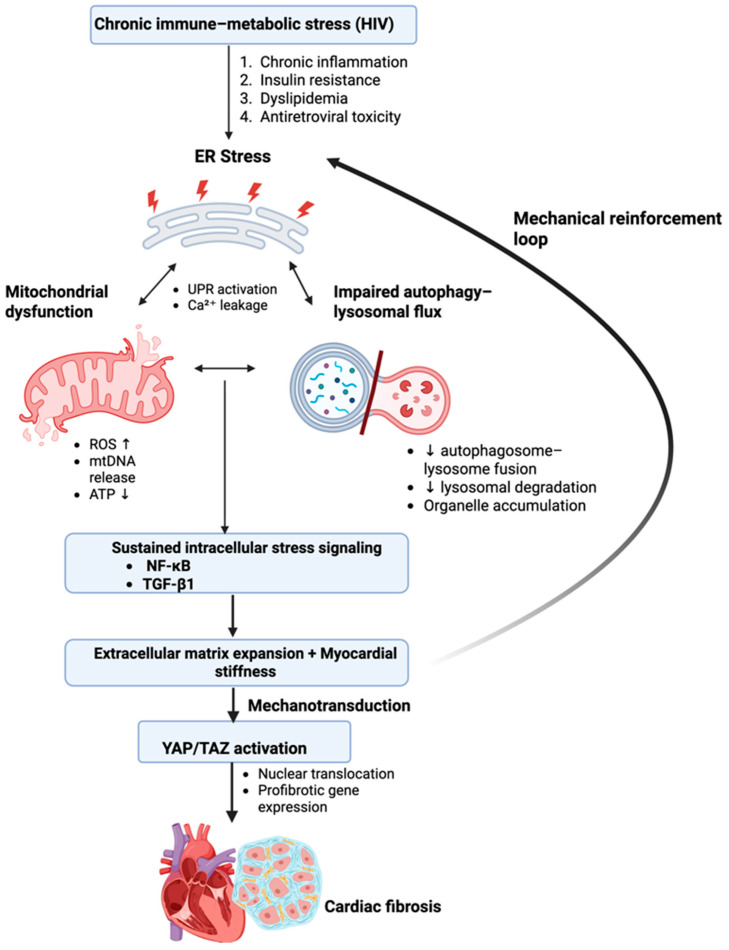
Integrated organelle stress network driving myocardial fibrosis in HIV. Chronic immune-metabolic stress in people with HIV initiates coordinated dysfunction across cardiomyocyte organelles, including mitochondria, the endoplasmic reticulum (ER), and the autophagy–lysosomal system. These organelles form an interdependent network in which mitochondrial reactive oxygen species (ROS) production, ER calcium dysregulation, and impaired autophagic clearance amplify one another. This unresolved organelle stress sustains intracellular inflammatory and profibrotic signaling, including nuclear factor kappa B (NF-κB) and transforming growth factor beta 1 (TGF-β1) activation, promoting extracellular matrix accumulation and myocardial stiffness. Increased tissue stiffness further activates mechanotransduction pathways, including Yes-Associated Protein and Transcriptional co-Activator with PDZ-binding motif (YAP/TAZ), leading to sustained profibrotic gene expression. A mechanical reinforcement loop between tissue stiffness and organelle stress stabilizes myocardial fibrosis. Created in BioRender. Masenga, S. (2026) https://BioRender.com/2u0xpe3.

The failure to resolve organelle injury represents a critical tipping point. Accumulated mitochondrial damage sustains redox-sensitive inflammatory pathways, ER stress reinforces maladaptive transcriptional responses, and defective autophagy locks cardiomyocytes into a state of chronic intracellular stress. Together, these processes converge on canonical profibrotic signaling pathways that promote fibroblast activation, collagen synthesis, and extracellular matrix expansion (Figure 4).

As extracellular matrix accumulates, myocardial stiffness increases, altering mechanical load and further feeding back into stress-sensitive signaling networks within cardiomyocytes and fibroblasts. In this stiffened microenvironment, mechanosensitive signaling through the YAP/TAZ axis becomes activated, promoting nuclear translocation of YAP/TAZ and sustained transcription of profibrotic genes [10,11,37,38]. Notably, these events unfold well before overt systolic dysfunction or clinically apparent cardiomyopathy, providing a mechanistic explanation for the high burden of diffuse myocardial fibrosis and diastolic dysfunction observed in people with HIV despite preserved ejection fraction and effective viral suppression [23,24].

This convergence model reframes HIV-associated myocardial fibrosis as the cumulative outcome of unresolved organelle stress rather than a late-stage consequence of structural heart disease. By positioning mitochondrial, ER, and autophagic dysfunction within a single integrated pathway, it highlights subcellular homeostasis as both a mechanistic nexus and a potential therapeutic target.

## 3. Detecting Mitochondrial Dysfunction in HIV-Associated Myocardial Remodeling: Imaging and Circulating Biomarkers

Mitochondrial dysfunction plays a central role in the pathogenesis of myocardial remodeling and fibrosis, yet direct assessment of mitochondrial health in vivo remains challenging. Unlike structural abnormalities or systolic dysfunction, mitochondrial impairment is fundamentally a metabolic and bioenergetic disturbance, often preceding overt changes detectable by conventional cardiac imaging. In people with HIV, where myocardial injury evolves gradually under chronic immune-metabolic stress [3,4], the ability to identify early mitochondrial dysfunction is particularly important but remains incompletely developed.

From a physiological perspective, this challenge is not trivial. Only a minority of myocardial energy consumption is devoted to mechanical work, while the majority supports non-mechanical processes including ion homeostasis, substrate metabolism, and heat generation [39,40]. As a result, substantial mitochondrial dysfunction may occur without immediate changes in global cardiac performance, underscoring the need for sensitive metabolic and molecular readouts rather than reliance on late functional endpoints.

### 3.1. Advanced Imaging Approaches to Myocardial Energetics

Several imaging techniques have been developed to interrogate myocardial energy metabolism indirectly. Invasive approaches, such as measuring coronary sinus blood flow combined with arteriovenous oxygen content differences, have historically provided estimates of myocardial oxygen consumption but are unsuitable for routine clinical or research use, particularly in asymptomatic populations [41,42].

Non-invasive methods offer greater translational potential. Positron emission tomography (PET) using tracers such as carbon-11-labeled acetate or oxygen-15-labeled molecular oxygen enables the quantification of myocardial oxygen consumption and oxidative metabolism [43,44]. These techniques provide sensitive, real-time assessments of mitochondrial oxidative capacity and have demonstrated altered myocardial energetics in cardiometabolic disease. However, PET is limited by high cost, radiation exposure, short tracer half-life, and restricted availability, making it impractical for widespread use in HIV populations or resource-limited settings.

Phosphorus-31 magnetic resonance spectroscopy (^31^P-MRS) allows for the non-invasive assessment of myocardial high-energy phosphate metabolism, including ATP and phosphocreatine levels and creatine kinase flux [44,45]. These measurements provide direct insight into mitochondrial bioenergetic reserve and energetic coupling. Despite its mechanistic appeal, ^31^P-MRS suffers from low spatial resolution, technical complexity, and limited accessibility, restricting its clinical applicability [45]. Consequently, while PET and MRS offer valuable proof-of-concept data, they remain largely confined to specialized research settings.

### 3.2. Indirect Imaging Readouts Relevant to Mitochondrial Dysfunction

Given these limitations, attention has shifted toward imaging markers that, while not measuring mitochondrial function directly, reflect the downstream consequences of mitochondrial stress. Cardiac magnetic resonance techniques such as native T1 mapping and extracellular volume quantification capture diffuse interstitial expansion that arises from sustained profibrotic signaling linked to mitochondrial oxidative stress and energetic failure [46,47]. Similarly, speckle-tracking echocardiography detects early impairments in myocardial deformation [48,49], which may reflect altered energetics, calcium handling, and cytoskeletal integrity secondary to mitochondrial dysfunction.

In the context of HIV, these modalities provide clinically feasible surrogates of mitochondrial injury by identifying structural and functional manifestations of prolonged subcellular stress rather than attempting the direct measurement of mitochondrial metabolism [50,51].

### 3.3. Circulating Biomarkers of Mitochondrial Stress and Bioenergetic Disturbance

Circulating biomarkers represent an attractive complementary strategy for assessing mitochondrial dysfunction, particularly in large-scale or resource-limited settings. Traditional markers such as lactate, pyruvate, creatine kinase, and lactate-to-pyruvate ratios have been used to infer mitochondrial impairment but lack specificity and sensitivity, as they are influenced by multiple systemic processes [19].

More recently, stress-responsive biomarkers such as growth differentiation factor-15 and fibroblast growth factor-21 have gained interest as indicators of mitochondrial stress and metabolic dysregulation [52,53]. These proteins are upregulated in response to oxidative stress, impaired fatty-acid oxidation, and mitochondrial injury, linking them mechanistically to organelle dysfunction. However, their lack of cardiac specificity and overlap with generalized metabolic and inflammatory states limit their standalone diagnostic value in HIV.

Emerging approaches aim to improve specificity by focusing on biomarkers more directly tied to mitochondrial processes, including circulating mitochondrial DNA fragments and lipid intermediates such as acylcarnitines that reflect incomplete β-oxidation [19,54]. Multi-omics strategies integrating metabolomics, proteomics, and inflammatory profiling [55] offer further promise by capturing composite signatures of mitochondrial stress rather than relying on single markers.

### 3.4. Relevance to HIV-Associated Myocardial Fibrosis

In people with HIV, mitochondrial dysfunction arises from the combined effects of antiretroviral therapy, dyslipidemia, insulin resistance, and chronic immune activation [5]. Biomarkers and imaging readouts that capture mitochondrial stress therefore reflect not only energetic impairment but also the upstream immune-metabolic milieu that drives organelle dysfunction and downstream fibrotic remodeling. Importantly, these measures may identify myocardial vulnerability at a stage when structural fibrosis is still diffuse and potentially modifiable.

Together, the current evidence highlights both the promise and limitations of existing tools for detecting mitochondrial dysfunction. While the direct measurement of mitochondrial energetics remains largely confined to research settings, clinically accessible imaging and biomarker approaches can capture the biological consequences of mitochondrial stress. Integrating these modalities within an organelle-centered framework may provide a pragmatic path toward early detection, mechanistic phenotyping, and targeted intervention in HIV-associated myocardial remodeling.

## 4. Therapeutic and Translational Implications: Restoring Organelle Homeostasis to Limit Myocardial Fibrosis in HIV

Reframing HIV-associated myocardial fibrosis as the cumulative consequence of unresolved organelle stress has important therapeutic implications. Rather than targeting fibrosis only after structural remodeling is established, this framework highlights earlier intervention points aimed at restoring mitochondrial function, alleviating endoplasmic reticulum stress, and improving autophagic clearance. Such strategies may interrupt profibrotic signaling before extracellular matrix expansion becomes self-sustaining.

### 4.1. Targeting Mitochondrial Dysfunction and Oxidative Stress

Given the central role of mitochondrial injury in initiating profibrotic signaling, therapies that improve mitochondrial efficiency or reduce oxidative stress represent rational upstream interventions. Optimization of metabolic control, including lipid lowering and insulin sensitization, may reduce substrate overload and limit mitochondrial ROS generation, thereby attenuating downstream profibrotic signaling [56,57]. Pharmacologic agents with mitochondrial effects, such as antioxidants targeted to mitochondria or modulators of mitochondrial biogenesis, have shown promise in preclinical models [58,59,60], although clinical translation in HIV remains limited.

Beyond systemic metabolic interventions, several pharmacologic strategies have been developed to directly target mitochondria. Mitochondria-targeted antioxidants aim to neutralize ROS at their primary source rather than relying on nonspecific cytosolic scavenging. Coenzyme Q10 (CoQ10), an essential component of the electron transport chain with intrinsic antioxidant properties, has been shown to improve mitochondrial redox balance and regenerate endogenous antioxidants [58]. Clinical studies in chronic heart failure have demonstrated improvements in symptoms and reductions in major adverse cardiovascular events, supporting the concept that mitochondrial redox modulation can influence cardiac outcomes [61].

To overcome variable cellular uptake, CoQ10 and other antioxidants have been conjugated to lipophilic cations such as triphenylphosphonium, enabling preferential accumulation within mitochondria driven by the negative mitochondrial membrane potential [58]. The resulting compound, mitoquinone (MitoQ), has shown efficacy in preclinical models, where it reduces oxidative stress, attenuates hypertension, cardiac hypertrophy, and improves vascular function [62]. Although clinical experience remains limited, these studies provide proof of principle that selective mitochondrial targeting is feasible.

Additional approaches focus on stabilizing mitochondrial membranes and preserving bioenergetic function. Elamipretide (SS-31) is a mitochondria-targeted peptide that localizes to the inner mitochondrial membrane and interacts with cardiolipin, thereby improving electron transport efficiency and reducing ROS generation [63,64]. While preclinical models of heart failure demonstrated the reversal of mitochondrial dysfunction and improved cardiac performance, early clinical trials have yielded mixed results [58], highlighting ongoing challenges in translating mitochondrial therapies to human disease.

Modulation of mitochondrial permeability transition also represents a mechanistically relevant strategy. Agents such as cyclosporine A inhibit cyclophilin D and prevent opening of the mitochondrial permeability transition pore, a key event linking mitochondrial stress to cell injury [58,59]. Although clinical benefits have been inconsistent, these studies underscore the therapeutic relevance of mitochondrial signaling pathways in cardiac pathology.

In the context of HIV, translation of mitochondrial-targeted therapies remains limited. However, chronic exposure to immune-metabolic stress and antiretroviral-related mitochondrial toxicity suggests that this population may be particularly susceptible to interventions that restore mitochondrial homeostasis. Importantly, antiretroviral therapy selection and optimization may themselves influence mitochondrial health. Minimizing cumulative mitochondrial toxicity and adverse metabolic effects could reduce long-term myocardial vulnerability, especially in aging people with HIV.

### 4.2. Modulating ER Stress and Proteostasis

Experimental studies provide proof of principle that the attenuation of ER stress can limit cardiac fibrotic remodeling [65]. Chemical chaperones that enhance protein-folding capacity and restore proteostasis have shown consistent antifibrotic effects in preclinical models [66]. In rodent models of cardiac fibrosis induced by β-adrenergic stimulation or angiotensin II exposure, pharmacologic reduction in ER stress using 4-phenylbutyric acid was associated with reduced expression of ER stress markers and the significant attenuation of myocardial collagen deposition [66]. Importantly, suppression of ER stress in these models was accompanied by the downregulation of profibrotic mediators, supporting a causal role for ER stress in sustaining fibrotic remodeling. Although direct ER-targeted therapies have not yet been translated into routine cardiovascular practice, these findings demonstrate that ER stress is a modifiable contributor to myocardial fibrosis. At present, chemical chaperones such as 4-phenylbutyric acid remain experimental, and their long-term safety and efficacy in cardiac disease require further evaluation.

In the context of HIV, where chronic immune activation, metabolic dysregulation, and mitochondrial dysfunction impose sustained proteostatic stress, indirect strategies to alleviate ER stress may be particularly relevant. Interventions that reduce lipid overload, oxidative stress, or mitochondrial injury are likely to secondarily relieve ER stress and limit maladaptive remodeling. As with mitochondrial-targeted therapies, ER-focused interventions remain an emerging area, but existing preclinical evidence supports proteostasis restoration as a plausible antifibrotic strategy in HIV-associated myocardial disease.

### 4.3. Targeting Autophagic-Lysosomal Dysfunction as a Therapeutic Strategy

Autophagy plays a central role in maintaining cardiomyocyte homeostasis by clearing damaged organelles and protein aggregates [31,32]. While basal autophagy is generally protective, accumulating evidence indicates that the dysregulation of autophagic flux, particularly a mismatch between autophagosome formation and lysosomal degradation, can contribute to myocardial injury and remodeling in HIV infection [35]. Importantly, this distinction has therapeutic implications: interventions aimed at restoring effective autophagic clearance may be beneficial, whereas nonspecific activation of autophagy may be harmful.

Experimental studies in cardiac disease demonstrate that impaired lysosomal degradation with continued autophagosome accumulation promotes cellular dysfunction and cardiomyocyte loss [67]. Conversely, selective modulation of autophagy-related pathways can attenuate myocardial injury, even beyond the acute phase of stress. Pharmacologic agents such as cardiac glycosides and sodium-glucose cotransporter 2 inhibitors have been shown to suppress maladaptive forms of autophagy-associated cell death and improve cardiomyocyte survival in preclinical models of ischemic and metabolic cardiac injury [68].

In the context of HIV, chronic immune-metabolic stress and mitochondrial dysfunction are likely to impose sustained demands on the autophagic–lysosomal system. Evidence suggests that autophagosome formation may remain active, while lysosomal function and autophagic flux become inefficient, leading to the persistence of damaged mitochondria and unresolved cellular stress [3,33,34]. Therapeutic strategies that enhance lysosomal capacity or normalize autophagic flux, rather than indiscriminately activating autophagy, may therefore represent a rational approach to limiting ongoing myocardial remodeling.

Although autophagy-targeted therapies are not yet established in clinical cardiovascular practice, emerging data indicate that the modulation of autophagic resolution pathways is feasible and biologically relevant [68]. In HIV-associated myocardial disease, restoring autophagic efficiency may complement mitochondrial- and ER-directed strategies by enabling the clearance of damaged organelles, reducing oxidative stress, and preventing the stabilization of profibrotic remodeling.

### 4.4. Interrupting Profibrotic Signaling and Mechanotransduction

Once extracellular matrix accumulation alters myocardial mechanics, fibrotic remodeling may become self-sustaining and less dependent on ongoing immune or metabolic injury. This transition marks a critical therapeutic inflection point. At this stage, strategies that directly reduce matrix burden, alter cell–matrix interactions, or limit fibroblast activation may be required to complement upstream organelle-directed interventions.

Established antifibrotic therapies, particularly modulation of the renin–angiotensin–aldosterone system, have demonstrated consistent benefits in limiting myocardial fibrosis across diverse cardiac conditions [19,69,70]. Beyond their hemodynamic effects, these agents reduce collagen synthesis, promote matrix turnover, and lower myocardial stiffness, thereby attenuating mechanically driven profibrotic signaling [69]. In this context, their therapeutic value may lie not only in preventing fibrosis initiation but also in destabilizing fibrosis once mechanical reinforcement has occurred.

Emerging approaches aimed at modifying tissue mechanics or fibroblast responsiveness further expand the therapeutic landscape. Interventions that reduce extracellular matrix cross-linking, alter integrin-mediated cell adhesion, or blunt fibroblast activation hold promise for interrupting fibrosis persistence [71]. Although direct targeting of mechanotransduction pathways remains largely experimental, these strategies underscore the importance of addressing the mechanical consequences of fibrosis rather than focusing exclusively on upstream inflammatory or metabolic triggers.

In HIV-associated myocardial disease, where early organelle stress initiates remodeling and tissue stiffness stabilizes progression, therapeutic strategies that combine metabolic optimization, organelle protection, and antifibrotic modulation may be necessary to prevent irreversible myocardial fibrosis. Targeting the mechanical phase of remodeling therefore represents a pragmatic and clinically relevant extension of organelle-centered interventions.

### 4.5. Exercise as a Non-Pharmacologic Strategy to Restore Mitochondrial Homeostasis

Lifestyle interventions, particularly aerobic exercise, represent a powerful and accessible strategy for modulating mitochondrial function and limiting myocardial remodeling [72]. In cardiovascular disease, mitochondrial biogenesis and mitochondrial dynamics are frequently impaired, contributing to energetic insufficiency, oxidative stress, and progressive fibrosis. Experimental and clinical studies consistently demonstrate that aerobic exercise improves myocardial mitochondrial content, ATP production, and overall bioenergetic efficiency, supporting its role as a biologically active intervention rather than a purely supportive measure [72,73].

Aerobic exercise has been shown to enhance mitochondrial biogenesis through the upregulation of key transcriptional regulators, including peroxisome proliferator-activated receptor gamma coactivator-1α and downstream nuclear respiratory factors [72]. These adaptations increase mitochondrial DNA replication, improve oxidative capacity, and enhance antioxidant defenses, thereby reducing mitochondrial ROS generation. Importantly, exercise-induced improvements in mitochondrial biogenesis translate into improved myocardial energy supply and functional resilience under metabolic stress.

Beyond increasing mitochondrial number, aerobic exercise also restores balance in mitochondrial dynamics. Cardiovascular disease is characterized by excessive mitochondrial fission and reduced fusion, leading to fragmented, dysfunctional mitochondria [72,73,74]. Exercise training has been shown to normalize the expression of mitochondrial fusion proteins and limit pathological fission, thereby improving mitochondrial network integrity and efficiency [74]. Restoration of balanced fusion–fission dynamics is associated with reduced myocardial fibrosis, improved diastolic function, and enhanced resistance to stress-induced injury [75]. Supporting this concept in humans, recent three-dimensional ultrastructural analyses of skeletal muscle biopsies demonstrate that aging is associated with disrupted mitochondrial architecture and reduced mitofusin-2 (MFN2) expression, changes that are partially reversed by exercise training, highlighting MFN2-mediated restoration of the mitochondrial structure as a conserved mechanism linking physical activity to improved mitochondrial health [76]. Given the overlap between aging-related and HIV-associated mitochondrial stress, these findings provide a plausible structural framework through which exercise may mitigate organelle dysfunction in people with HIV.

In people with HIV, where chronic immune activation, metabolic dysregulation, and antiretroviral therapy contribute to mitochondrial stress, exercise may offer unique benefits. By simultaneously improving insulin sensitivity, lipid handling, and mitochondrial quality control, aerobic exercise addresses multiple upstream drivers of organelle dysfunction. Importantly, exercise represents a low-cost, scalable intervention that can be implemented across diverse clinical settings, including resource-limited environments where advanced pharmacologic therapies may not be readily available.

Although optimal exercise prescriptions for targeting myocardial mitochondrial health in HIV remain to be defined, existing evidence supports aerobic exercise as a safe and biologically plausible strategy to restore mitochondrial homeostasis, complement pharmacologic interventions, and potentially slow the progression of organelle-driven myocardial fibrosis.

### 4.6. Biological Variability: Sex, Age, and Mitochondrial Heterogeneity

Emerging clinical trials of mitochondria-targeted therapies highlight important gaps in the evaluation of biological variability. In the MMPOWER-3 trial, which included a relatively high proportion of women (64%) and a broad age range, elamipretide was well-tolerated but did not improve primary functional outcomes [77]. Notably, despite this demographic diversity, sex-specific analyses were not reported, limiting insight into potential differences in mitochondrial response between men and women. Similarly, while age-related variation was not a primary focus, pharmacokinetic analyses suggested that mitochondrial drug handling may vary with age, reflecting underlying differences in mitochondrial function and metabolic capacity. Importantly, post hoc analyses demonstrated differential responses based on genetic subtypes, with signals of benefit observed in participants with nuclear DNA-related mitochondrial defects but not in those with mitochondrial DNA alterations. These findings suggest that therapeutic responsiveness may depend more on underlying mitochondrial biology than on clinical phenotype alone. Critically, such trials have not been conducted in people with HIV, where chronic immune activation, antiretroviral exposure, and metabolic dysregulation may uniquely influence mitochondrial function and therapeutic response. Collectively, current evidence underscores a critical gap in precision stratification, where sex, age, and molecular heterogeneity remain under-integrated in the design and interpretation of organelle-targeted therapies, particularly in HIV-associated disease.

### 4.7. Implications for Resource-Limited, High-Burden Settings

In high HIV-burden and resource-limited settings, direct assessment of mitochondrial or subcellular dysfunction is often impractical. However, an organelle-centered framework highlights opportunities to detect early myocardial remodeling using accessible tools that capture the downstream consequences of organelle stress rather than organelle dysfunction itself. Speckle-tracking echocardiography offers a scalable, non-invasive approach to identify subtle abnormalities in myocardial deformation that may precede overt cardiomyopathy. When combined with selected circulating biomarkers reflecting immune activation, metabolic stress, or extracellular matrix turnover, this strategy enables pragmatic risk stratification and early intervention. By emphasizing early detection and prevention over late-stage treatment, this framework aligns with the realities of HIV care in resource-limited settings and supports targeted intervention before myocardial fibrosis becomes irreversible.
**Highlights and Future Directions** **Highlights**•HIV-associated myocardial fibrosis emerges from early, interconnected dysfunction of mitochondria, the ER, and lysosomal-autophagy pathways rather than from late-stage structural heart disease alone.•Chronic immune-metabolic stress initiates subcellular maladaptation that precedes detectable changes in cardiac function or conventional imaging.•Organelle stress converges on profibrotic remodeling through sustained extracellular matrix accumulation and mechanically reinforced signaling, stabilizing fibrosis even in virologically suppressed individuals.•Accessible tools, including speckle-tracking echocardiography and selected circulating biomarkers, provide pragmatic downstream readouts of organelle-driven myocardial injury.**Future Directions**•Longitudinal studies integrating imaging, biomarkers, and metabolic profiling are needed to define early trajectories of organelle-driven myocardial remodeling in people with HIV.•Therapeutic strategies targeting mitochondrial health, ER stress resolution, autophagic efficiency, and tissue mechanics warrant evaluation in HIV-specific populations.•Resource-adapted screening and prevention frameworks should be developed to identify high-risk individuals before irreversible myocardial fibrosis develops.

## 5. Conclusions

HIV-associated myocardial fibrosis reflects a shift from adaptive cellular stress responses to persistent subcellular maladaptation driven by chronic immune-metabolic burden. By framing cardiac remodeling as the cumulative outcome of unresolved mitochondrial, ER, and autophagic dysfunction, this review highlights organelle homeostasis as a unifying mechanistic nexus linking HIV to fibrotic heart disease. Importantly, this perspective reorients clinical and research priorities toward early detection and prevention, emphasizing scalable imaging and biomarker strategies capable of identifying myocardial vulnerability before irreversible fibrosis ensues. An organelle-centered framework therefore provides not only mechanistic insight but also a practical foundation for developing targeted, context-appropriate interventions to mitigate cardiovascular risk in people living with HIV.

## Figures and Tables

**Table 1:** Rationale and conceptual novelty of an organelle-centered framework for myocardial fibrosis in HIV.

Unmet Need/Conceptual Gap	Organellar Perspective Advanced in This Review
Subcellular origins of myocardial fibrosis are insufficiently defined	Positions mitochondrial dysfunction, ER stress, and lysosomal–autophagy impairment as early initiators of profibrotic signaling preceding overt cardiac dysfunction
2. The role of organelle cross-talk in sustaining fibrosis is underexplored	Highlights coordinated mitochondrial–ER–lysosomal interactions that reinforce a feed-forward cycle of metabolic stress, inflammation, and extracellular matrix expansion
3. Early detection of subclinical myocardial remodeling remains challenging	Links organelle dysfunction to mechanistically informed biomarkers and advanced imaging markers capable of detecting preclinical fibrosis
4. Organelle-targeted therapeutic strategies in HIV are undefined	Identifies translational opportunities to restore organelle homeostasis using metabolically focused and antifibrotic interventions relevant to HIV populations

## Data Availability

No new data were created or analyzed in this study. Data sharing is not applicable to this article.
